# The Moderating Role of Lifestyle on Insomnia in Home Quarantine College Students During the COVID-19 Epidemic

**DOI:** 10.3389/fpsyt.2022.830383

**Published:** 2022-03-02

**Authors:** Jinfang Zhang, Lin Mi, Jingbo Zhao, Huilin Chen, Dongfang Wang, Zijuan Ma, Fang Fan

**Affiliations:** ^1^School of Psychology, South China Normal University, Guangzhou, China; ^2^The Affiliated Brain Hospital of Guangzhou Medical University, Guangzhou, China; ^3^Department of Psychology, School of Public Health, Southern Medical University, Guangzhou, China; ^4^Department of Psychology, University of Bath, Bath, United Kingdom

**Keywords:** insomnia, lifestyle, college student, the COVID-19 epidemic, food intake

## Abstract

There has been sufficient evidence for the relationship between lifestyle and insomnia in the general population, but for individuals who already suffer from insomnia, it is not clear whether a healthy lifestyle can also pose similar benefits. The present study investigated the roles of different aspects of lifestyle in the development of individual insomnia by tracking insomnia symptoms of college students during the COVID-19 lock-down. Two surveys were conducted on 65,200 college students in the process of home isolation in Guangdong Province of China, at the pandemic outbreak period (T1) and the initial remission period (T2), respectively. Given the objectives of the present study, a total of 1,702 college students with clinical insomnia from T1 were selected as subjects. Insomnia symptoms were assessed using the Youth Self Rating Insomnia Scale (YSIS), while demographic information, epidemic exposure, and lifestyle were all measured by self-developed questionnaire, through network survey. The 1,702 college students (mean age ± standard deviation, 20.06 ± 1.46, range 16–25; 71.9% females) with insomnia symptoms were divided into three trajectory groups: recovery group, remission group, and chronic insomnia group according to their insomnia scores in T2 phase. The results showed that there was no significant difference in demographic backgrounds or epidemic exposure among the three groups, however, there were significant differences in food intake, exercise, and Internet use. The regression results further showed that both the recovery group and the remission group adopted more regular food intake than the chronic group. The recovery group exhibited better daily exercise habits than both the remission group and the chronic group. The duration of Internet use was significantly shorter for the recovery group than for the chronic group. These findings indicate a strong relationship between the lifestyle and the recovery of insomnia for college students isolated at home during the epidemic period. Significance of the different aspects of lifestyle on the recovery of insomnia are discussed.

## Introduction

Previous studies indicated that college students were prone to sleep disruption (1–5). A meta-analysis among college students reported the pooled mean prevalence of insomnia of 18.5% (9.4–38.2%) (6), which was higher than that in general population in Italy (7.4%) (7), as well as that among the general population in China (9.2%) (8). A recent study on Norwegian college students found a substantial increase in sleep problems from 2010 (22.6%) to 2018 (30.5%) (5). Given the already high prevalence, such upward trend is a concern needing to be addressed.

Insomnia exerts non-negligible adverse effects on individual's daily life. It is reported that insomnia not only impairs physical and mental functions and reduces work productivity, but could also cause mental problems, such as anxiety and depression, and even suicide (9–15). The primary aim of the present study was therefore to find the factors associated with the recovery from insomnia in college students.

Lifestyle has received increasing attention in medicine in recent years (16). Lifestyle changes can be beneficial for preventing, treating, and even reversing the progression of chronic diseases by addressing their underlying causes (17). Emerging evidence have revealed relationships between different aspects of lifestyle and insomnia, such as food intake (18, 19), exercise (20–22), and Internet use (23, 24).

The relationship between food intake and sleep has been an important research question. First of all, from the perspective of nutritional intake, dietary tryptophan can directly affect sleep quality (25). More importantly, there is emerging evidence that the food intake regularity with regards to amount is correlated with sleep disturbance. For example, overeating, especially binge eating disorder, is associated with insomnia symptoms. Individuals with binge eating disorder reported more severe insomnia symptoms than individuals without a history of binge eating disorder (19, 26). It is also demonstrated in a four-year follow-up study that excess food intake induced poor sleep quality (27). On the other hand, there are also findings which support the hypothesis that the food intake regularity with regards to time is correlated with sleep disturbance. In a cross-sectional study, the timing of meals during COVID-19 home isolation was associated with sleep disturbances (28). Adults with insomnia had more nighttime eating habits than those without insomnia (29). An individual's nighttime eating habits may lead to delays in their biorhythms, which in turn may delay sleep phases (30). On the basis of the existing studies, the current study focused on whether the regularity of food intake could help regulate sleep patterns in individuals with insomnia.

Exercise is often thought to promote sleep and reduce insomnia symptoms and is recommended as a non-pharmacological treatment in insomnia treatment guidelines (20, 31). However, empirical findings have not always been consistent. In terms of intervention for insomnia and related symptoms, most RCT studies have shown that specific form and patterns of exercise, especially aerobic exercise and exercising at regular intervals, can promote better sleep quality and thus alleviate symptoms of insomnia (32–35). These studies are usually designed with a blank control group (i.e., no training task was employed for the control group), achieving moderate or higher effect sizes. However, when individuals who completed health education was employed for the control group, no gain of exercise was found, or only very limited positive effect (36). Thus, the relationship between exercise and insomnia symptoms will be further explored in the current study in a longitudinal investigation.

China is reported to have the largest number of Internet users in the world, with an estimation of 883 million Internet users in 2019, which is estimated to surge to 1.14 billion by 2025 (37). Having become an important part of daily life, Internet use is bound to have an important and long-term impact on users' mindsets and behaviors. Cross-sectional investigation on the use of Internet and related electronic products found that excessive or even addictive Internet use were associated with mental and psychological problems, including sleep problems such as insomnia (38, 39). A few longitudinal studies have examined the causal relationship between the two. An 8-month follow-up survey of Chinese vocational school students reported that excessive Internet use was a contributor to insomnia (40). At present, most studies focus on sleep problems and insomnia from the perspective of Internet addiction and problematic Internet use. The relationship between non-excessive Internet use and insomnia remains unclear. In the case of home quarantine during the outbreak of COVID-19, the Internet was predominantly the only way for individuals to connect with the outside world, which may have a different meaning for an individual's sleep than what it used to have.

### Present Study

The outbreak of COVID-19 is a serious public health crisis with extensive psychological and behavioral consequences. During the SARS outbreak in 2003, the proportion of people who reported insomnia increased significantly (41, 42). In the wake of the COVID-19 outbreak, studies have reported rates of insomnia ranging from 7.3 to 37.6% (43–47), greater than the pre-pandemic worldwide insomnia prevalence, estimated before the pandemic between 3.9 and 22% (48). An Italian study that compared insomnia symptoms among 240 college students with both gender and age controlled, before and after the pandemic, found that college students experienced more severe insomnia symptoms during the quarantine period than before the pandemic (49). In China, in order to combat the pandemic more effectively, home quarantine was implemented, which reduced the influence from the outside world. It was demonstrated that irregular lifestyle in food intake, exercise, and Internet use in home quarantine individuals during the epidemic placed great pressure on physical and mental health (50–54). Studies have found significant changes in eating habits during the pandemic, and some individuals may increase their food intake to alleviate negative emotions (50). Similarly, physical exercise habits are difficult to maintain during home isolation (51). Maintaining good exercise habits improves mental health (52), and more exercise reduces the incidence of insomnia (53). A study finds that students showed more changes in their routine behaviors than non-students after the outbreak, and insomnia scores increased more than that of non-students (54); Many studies have reported increased time spent using Internet and related electronic devices under isolation and outdoor activity restrictions (55, 56), and a strong relationship was found between bedtime use of electronic devices and sleep problems (57, 58). On other hand, the maintenance of a healthy life style was shown to reduce the adversity of physical problems and maintaining physical and mental health (59, 60). Taken together, behavior management with an aim to maintain the stability of different aspects of lifestyle might be of great clinical values.

The present study thus aimed to explore the factors that are protective against insomnia from the perspective of lifestyle. We hypothesized that a healthy lifestyle would be beneficial for overcoming insomnia among college students who were isolated at home. Most of the existing studies use cross-sectional design, which limited their capacity to explore the temporal role of lifestyle in the prognosis of insomnia. Therefore, the current study was designed to examine the relationship between lifestyle and insomnia from the perspective of the dynamic evolution of sleep problems.

## Materials and Methods

### Samples and Design

This study was part of a school-based cohort study. The baseline and follow-up of this study were conducted from February 3 to February 10, 2020 (shortly after the pandemic outbreak, T1), and from March 24th to April 3rd, 2020 (before the pandemic over when the epidemic began to remiss, T2), respectively. A total of 65,200 undergraduate students under home quarantine in Guangdong Province, China, completed both self-report surveys. Details of sample characteristics in both surveys were described elsewhere (61, 62).

A general questionnaire was designed to collect information on socio-demographics, exposure with COVID-19 epidemic, psychosocial factors and lifestyle. Surveys were conducted through the network platform (“http://www.togx.cn/step_50.html”). The investigation was approved by the Human Research Ethics Committees of South China Normal University (Ethics_No._SCNU-PSY-2020-01-001). All participants were given electronic informed consent before starting the online survey. Participations in this study were entirely voluntary and were informed that they could quit the experiment at any time.

### Measures

Socio-demographic variables including gender, age, numbers of children in family, living in a rural area or urban area, were collected by dichotomous or ordinal questions at baseline (T1).

Three items were developed to assess individual exposure to COVID-19 (1) infected cases with COVID-19 in the community or village (0 = No, 1 = Yes); (2) relatives or acquaintances being contracted with COVID-19 (1 = Nobody, 2 = Do not know, 3 = Confirmed or Suspected); (3) Pandemic severity in the living province (1 = Mild, 2 = Moderate/ Severe). The severity of the province in the epidemic exposure was derived from the T1 measurement. A combination of two measurements (T1 and T2) was used to assess the extent of community outbreaks and relatives or acquaintances infections.

Lifestyle was measured from three aspects: food intake, daily exercise, and Internet use in T2. The survey question about food intake regularity was: “*In the past two weeks, have you been eating three meals a day regularly on aspects of time and amount?”* The subjects were asked to respond on a four-point scale (0 = Never, 1 = Seldom, 2 = Sometimes, 3 = Always). Daily exercise question was: “*In the past two weeks, how much time did you spend on average exercising every day?”* Answers were also recorded on a four-point scale (0 = Never, 1 = <30 min, 2 = Between 30 and 60 min, 3 = More than 60 min). The question about Internet use was: “*In the past two weeks, how much time did you use the Internet every day?”* Answers were recorded on a three-point scale (0 = ≤2 h, 1 = Between 3 and 5 h, 2 = More than 5 h).

Insomnia was evaluated using the Youth Self Rating Insomnia Scale (YSIS), which is a self-rating insomnia scale for Chinese adolescents (63), with an internal consistency reliability coefficient of 0.80, and a retest reliability coefficient of 2 weeks of 0.82. In the present sample, Cronbach's α for the total score were 0.89 and 0.90, respectively, at two time points. The YSIS has 8 items and evaluates 3 aspects: (1) Insomnia symptoms; (2) Self-awareness of sleep quality; (3) The effect of insomnia on daily function. Answers were scored on a 5-point Likert scale, with a total score of 40. Higher scores implied more severe insomnia. The cutoffs of the insomnia score were as follows: Normal, <22; Mild insomnia, 22–25; Moderate insomnia/probable clinical insomnia, 26-29; Severe insomnia/clinical insomnia, ≥30. Moderate insomnia and severe insomnia were pooled together, and categorized as clinic insomnia.

### Statistical Analysis

All analyses were conducted with SPSS 22.0 for Windows in this study (IBM SPSS Statistics). Socio-demographic variables were first calculated descriptively. 1,702 subjects who reached probable clinical level of insomnia level at T1 were divided into recovery group (<22), remission group (22–25) and chronic group (≥26), according to the trajectory of insomnia severity as measured by YSIS between T1 and T2. Chi-square tests were performed for between-group differences in categorical variables such as demography, pandemic exposure, and living habits, and *T*-test or ANOVA for continuous variables. Moreover, multiple logistic regression was conducted to examine the predictive factors associated with insomnia trajectory membership. Age, insomnia score at T1 was treated as continuous, while other predictors were treated as categorical. Outcomes of two-sided tests with *P*-value <0.05 were regarded as being statistically significant.

## Results

### Demographic Characteristics, the COVID-19 Exposure, and Psychosocial Measurements by Insomnia Status

In the outbreak period, the incidence of insomnia symptoms in college students isolated at home was 8.6%, and the incidence of clinical insomnia reached 2.6%. There were significant differences among the three groups in epidemic exposure and demographic characteristics except for age (see Table 1).

**Table 1:** Demographics and the COVID-19 exposure of the total sample.

**Variable**	**No insomnia** **(*n =* 59584)**	**Mild insomnia** **(*n =* 3914)**	**Clinic insomnia** **(*n =* 1702)**	***F*/χ^2^**	***P***
**Gender**					
Male	18,936 (31.8%)	1,118 (28.6%)	479 (28.1%)	27.15	0.000
Female	40,648 (68.2%)	2,796 (71.4%)	1,223 (71.9%)		
Age (*M, sd*)	20.09 (1.49)	20.11 (1.49)	20.06 (1.46)	0.64	0.527
**No. of children in the family**					
1	11,954 (20.1)	841 (21.5%)	405 (23.8%)	17.68	0.000
≥2	47,630 (79.9)	3,073 (78.5%)	1,297 (76.2%)		
**Location**					
Rural	24,468 (41.1%)	1,325 (33.9%)	521 (30.6%)	152.09	0.000
Urban	35,116 (58.9%)	2,589 (66.1%)	1,181 (69.4%)		
**Severity in the living province**					
Mild	5,108 (8.6%)	288 (7.4%)	135 (7.9%)	7.95	0.019
Moderate/ Severe	54,476 (91.4%)	3,626 (92.6%)	1,567 (92.1%)		
**Infection in community or village**					
No	53,852 (90.4%)	3,378 (86.3%)	1,403 (82.4%)	153.65	0.000
Yes	5,732 (9.6%)	536 (13.7%)	299 (17.6%)		
**Infection in relative or acquaintances**					
No	33,194 (55.7%)	1,689 (43.2%)	687 (40.4%)	390.96	0.000
Do not know	25,046 (42.0%)	2,095 (53.5%)	933 (54.8%)		
Confirmed or suspected	1,344 (2.3%)	130 (3.3%)	82 (4.8%)		

### The Trend of Insomnia and the Difference of Lifestyle in Each Group

For 1,702 college students who fell into the insomnia group on the first test, there was a significant difference in their insomnia scores between T1 and T2 [29.02 ± 3.028 vs. 24.76 ± 6.486, *t* (1,701) = −27.299; *P* < 0.001; Cohen's *d* = 0.84], that is, the insomnia symptom of college students remitted significantly as a whole in the remission period than that in the outbreak period of COVID-19.

**Figure 1:**
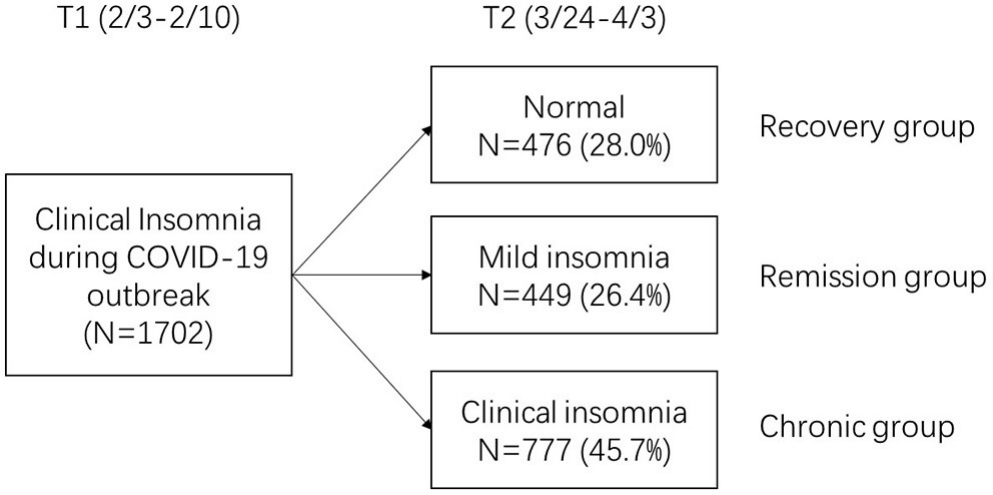
Trajectories of insomnia symptoms between two time points after the COVID-19 epidemic.

According to the classification of insomnia trajectories, by the time of the follow-up, 28% of the students recovered to normal sleep state (without insomnia), constituting of the recovery group; 26.4% of the students showed initial reduction in symptoms, with scores of T2 measures indicating mild insomnia (remission group). 45.7% of college students continued to show moderate to severe insomnia, namely chronic group (see Figure 1).

As seen in Table 2, Chi-square analysis results showed that there was no significant difference in demographic backgrounds among recovery group, remission group and chronic group, including gender, one-child status and family residence. In the investigation of pandemic exposure, there was no significant difference among the three groups in terms of the severity of the pandemic in the province where they lived, the infection situation in the community or village where they lived, and the infection situation of the close friends and relatives.

**Table 2:** Demographics, the COVID-19 exposure, lifestyle, and insomnia in recovery group, remission group and chronic group.

**Variable**	**Recovery** **(*n =* 476)**	**Remission** **(*n =* 449)**	**Chronic** **(*n =* 777)**	***F*/χ^2^**	***P***
**Gender**					
Female	339 (71.2%)	330 (73.5%)	554 (71.3%)	0.819	0.664
Male	137 (28.8%)	119 (26.5%)	223 (28.7%)		
Age (*M, sd*)	20.07 (1.401)	20.15 (1.479)	20.01 (1.49)	1.392	0.249
**No. of children in the family**					
1	105 (22.1%)	105 (23.4%)	195 (25.1%)	1.564	0.458
≥2	371 (77.9%)	344 (76.6%)	582 (74.9%)		
**Location**					
Rural	146 (30.7%)	142 (31.6%)	233 (30.0%)	0.360	0.835
Urban	544 (69.3%)	307 (68.4%)	544 (70.0%)		
**Severity in the living province**					
Mild	41 (8.6%)	37 (8.2%)	57 (7.3%)	0.740	0.691
Moderate/ Severe	435 (91.45)	412 (91.8%)	720 (92.7%)		
**Infection in community or village**					
No	398 (83.6%)	378 (84.2%)	627 (80.7%)	3.028	0.220
Yes	78 (16.4%)	71 (15.8%)	150 (19.3%)		
**Infection in relative or acquaintances**					
No	213 (44.7%)	183 (40.8%)	291 (37.5%)	8.751	0.068
Do not know	236 (49.6%)	247 (55.0%)	450 (57.9%)		
Confirmed or suspected	27 (5.7%)	19 (4.2%)	36 (4.6%)		
**Eating regularly**					
Never	16 (3.4%)	19 (4.2%)	83 (10.7%)	131.661	0.000
seldom	39 (8.2%)	41 (9.1%)	160 (20.6%)		
Sometimes	117 (24.6%)	151 (33.6%)	254 (32.7%)		
Always	304 (63.9%)	238 (53.0%)	280 (36.0%)		
**Exercise**					
Never	64 (13.4%)	81 (18.0%)	180 (23.2%)	39.169	0.000
<30 min	254 (53.4%)	249 (55.5%)	439 (56.5%)		
30–60 min	113 (23.7%)	96 (21.4%)	126 (16.2%)		
>60 min	45 (9.5%)	23 (5.1%)	32 (4.1%)		
**Internet use**					
≤2 h	30 (6.3%)	19 (4.2%)	29 (3.7%)	30.366	0.000
3–5 h	95 (20.0%)	87 (19.4%)	86 (11.1%)		
>5 h	351 (73.7%)	343 (76.4%)	662 (85.2%)		
YSIS (T1)	28.30 (2.61)	28.61 (2.80)	29.70 (3,24)	39.01	0.000

*Note: Severity in the living province, measured at T1; Infection in community or village, NO = answer “NO” both at T1 and T2, YES = answer “YES” at least once at T1 and T2; Infection in relative of acquaintances, NO = answer “NO” both at T1 and T2, Do not know = answer “Do not know” both at T1 and T2, Confirmed or suspected = answer “Confirmed or suspected” at least once at T1 and T2; YSIS (T1) = insomnia score at T1*.

**Figure 2:**
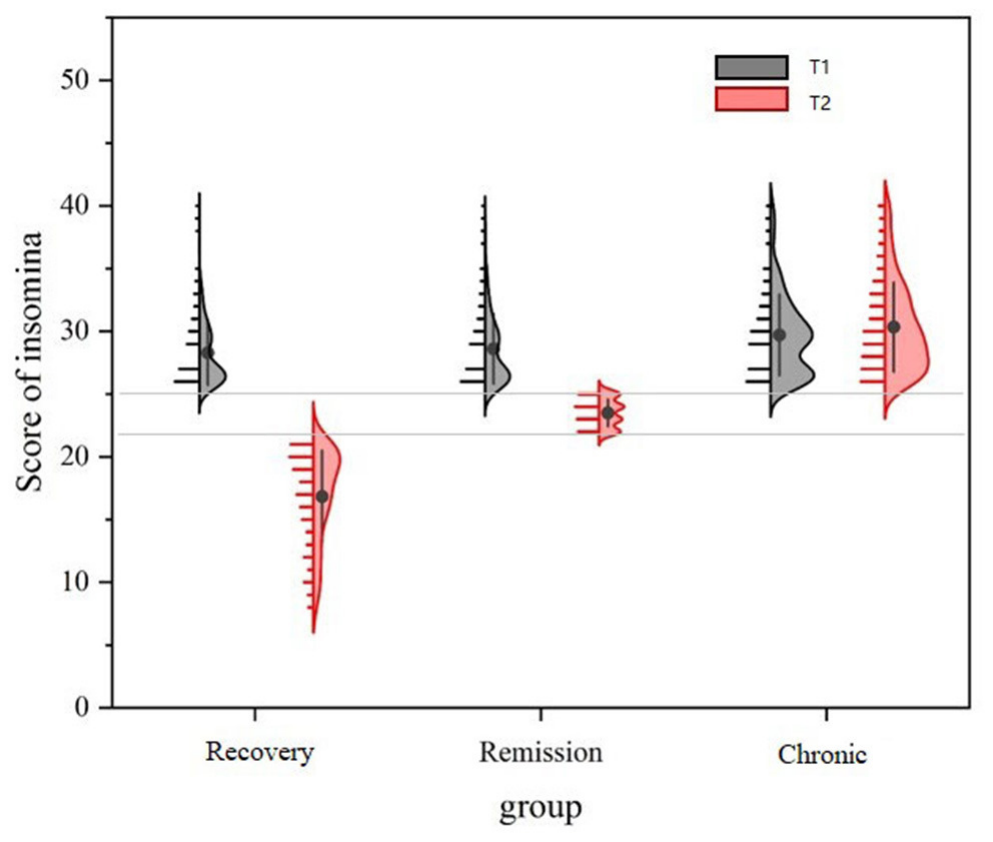
Scores of insomnia symptoms in recovery group, remission group and chronic group at T1 and T2.

The result of ANOVA showed that there were significant differences in insomnia scores between the three groups at T1 [*F*_(2,1,699)_ = 39.01; *P* < 0.0001; ${\eta_{p}}^{2}$ = 0.044]. Tukey *post-hoc* test indicated that the effect was mainly driven by the difference between the chronic group and recovery group (*P* < 0.0001), also by the difference between the chronic group and remission group (*P* < 0.0001) (see Figure 2). There were also significant differences among the three groups in food intake, exercise and Internet use.

**Figure 3:**
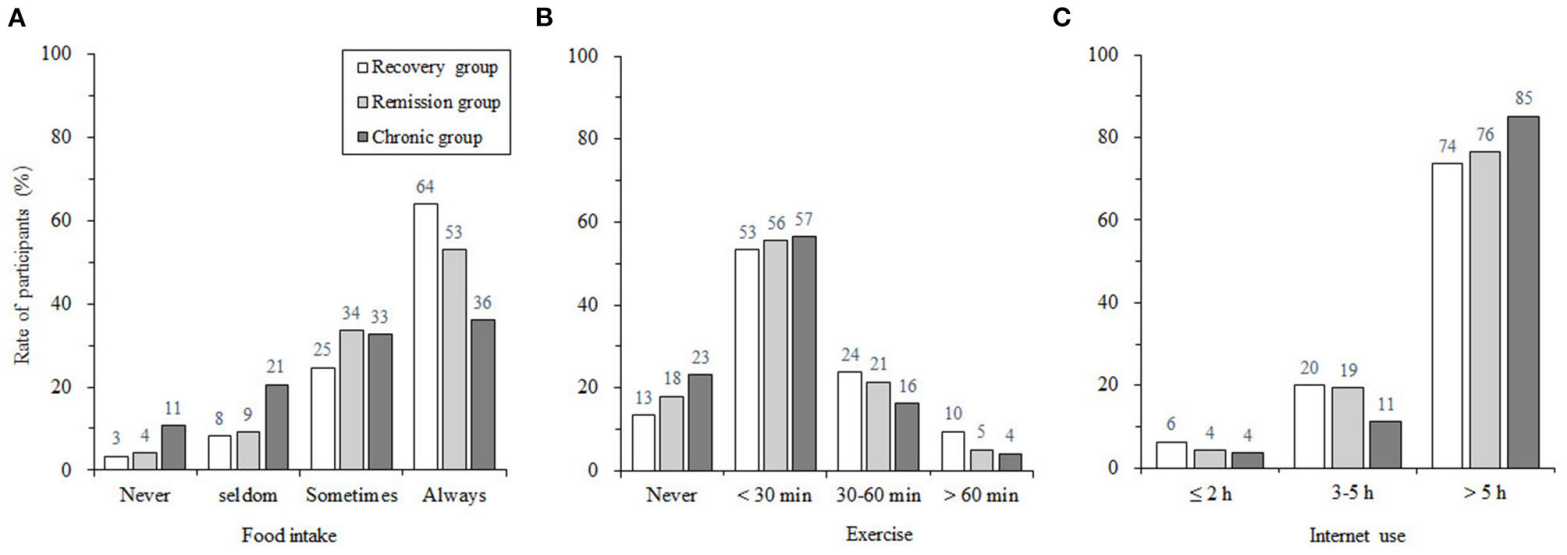
The proportion of participants in the aspect of food intake regularity **(A)**, exercise duration **(B)**, and the Internet use duration **(C)** for recovery group, remission group, and chronic group.

Significant between-group differences were found for participants with different insomnia trajectories on the three aspect of lifestyles (see Figure 3). In terms of food intake regularity, distribution patterns of eating regularity measures across the three groups of subjects were different. More specifically, the food intake distribution (from “*Never”* to “*Always”*) of the recovery group was most inclined to be regular, the remission group was second, and the chronic group was the worst. For daily exercise, more than half of the subjects in all three groups chose “*Less than 30 minutes”*. In terms of the differences between the groups, a higher percentage of people in the recovery group exercised for more than 30 min, and fewer did not exercise, while the chronic group showed the opposite pattern. In terms of Internet use time, the majority of subjects in all three groups chose “*More than 5 hours”*, more than 70%. Specifically, the proportion of people in the recovery group was the lowest, and the proportion of people in the chronic group was the largest.

### Logistic Regression of the Influence of Lifestyle on the Trajectory of Insomnia

Multivariate regression analysis was conducted to investigate the relationship between the changes in insomnia symptoms of the three groups of subjects, and food intake, exercise habits, and Internet use, controlling for the effects of epidemic exposure, demographic variables, and baseline insomnia level (see Table 3). Taking the recovery group as reference, multivariate logistic regression analyses were conducted. College students who chose “*Do not know”* as an answer to the infection in relative or acquaintances (OR for “*Do not know”* vs. “*Nobody”* being 1.33, 95% CI 1.03–1.71, *P* = 0.03), showed more severe symptoms of insomnia at T1 (OR 1.17, 95% CI 1.12–1.22, *P* < 0.001) and more Internet use (at T2) (OR for > 5 h vs. ≤ 2 h = 2.05, 95% CI 1.16–3.61, *P* = 0.01), with higher likelihood of developing chronic insomnia. In addition, individuals who reported less regular eating habits (OR for Sometimes vs. Never = 0.55, 95% CI 0.30–1.00, *P* = 0.05; OR for Always vs. Never = 0.25, 95% CI 0.14–0.45, *P* < 0.001) and had less time for exercise (OR for 30–60 min vs. Never = 0.65, 95% CI 0.43–0.98, *P* = 0.04; OR for > 60 min vs. Never = 0.35, 95% CI 0.20–0.62, *P* < 0.001) had a higher risk of chronic insomnia (see Table 3). Taking the recovery group as reference, college students in remission group reported less time for exercises (OR for > 60 min vs. Never = 0.43, 95% CI 0.23–0.79, *P* < 0.01). Taking the chronic group as reference, individuals with lower insomnia scores at T1 (OR 0.89, 95% CI 0.86–0.93, *P* < 0.001) and kept more regular eating habits (OR for Sometimes vs. Never = 2.28, 95% CI 1.30–3.99, *P* < 0.0001; OR for Always vs. Never = 3.09, 95% CI 1.78–5.36, *P* < 0.001) had a higher likelihood of insomnia remission.

**Table 3:** Multivariate logistic regression of predictors on insomnia trajectories.

**Variable**	**Remis vs. recovery**	**chronic vs. recovery**	**Remis vs. chronic**
	**OR (95% CI)**	**OR (95% CI)**	**OR (95% CI)**
**Gender**			
Male	1	1	
Female	1.11 (0.82–1.49)	0.97 (0.74–1.28)	1.14 (0.87–1.50)
Age	1.04 (0.95–1.14)	0.98 (0.90–1.06)	1.07 (0.98–1.16)
**No. of children in the family**			
1	1	1	
≥2	0.90 (0.65–1.25)	0.77 (0.57–1.05)	1.17 (0.86–1.16)
**Location**			
Rural	1	1	
Urban	1.00 (0.74–1.35)	1.03 (0.78–1.36)	0.97 (0.74–1.28)
**Severity in the living province**			
Mild	1	1	
Moderate/ Severe	1.00 (0.74–1.35)	1.230 (0.77–1.95)	0.81 (0.51–1.29)
**Infection in community or village**			
Yes	1	1	
No	1.12 (0.77–1.61)	1.02 (0.73–1.42)	1.09 (0.79–1.52)
**Infection in relative or acquaintances**			
No	1	1	
Do not know	1.20 (0.91–1.57)	**1.33 (1.03–1.71)** ^*^	0.90 (0.70–1.16)
Confirmed or suspected	0.81 (0.43–1.53)	0.76 (0.42–1.35)	1.07 (0.58–1.98)
**Eating regularly**			
Never	1	1	
Seldom	1.00 (0.44–2.25)	1.03 (0.53–2.00)	0.97 (0.52–1.18)
Sometimes	1.25 (0.60–2.58)	**0.55 (0.30–1.00)** ^†^	**2.28 (1.30–3.99)** ^**^
Always	0.76 (0.38–1.55)	**0.25 (0.14–0.45)** ^***^	**3.09 (1.78–5.36)** ^***^
**Exercise**			
Never	1	1	
<30 min	0.79 (0.54–1.15)	0.76 (0.54–1.08)	1.04 (0.75–1.43)
30–60 min	0.74 (0.47–1.15)	**0.65 (0.43–0.98)** ^*^	1.13 (0.76–1.69)
>60 min	**0.43 (0.23–0.79)** ^**^	**0.35 (0.20–0.62)** ^***^	1.23 (0.66–2.30)
**Internet use**			
≤2 h	1	1	
3–5 h	1.49 (0.78–2.87)	1.11 (0.59–2.09)	1.34 (0.68–2.65)
>5 h	1.54 (0.84–2.81)	**2.05 (1.16–3.61)** ^*^	0.75 (0.41–1.40)
YSIS (T1)	1.04 (0.99–1.10)	**1.17 (1.12–1.22)** ^***^	**0.89 (0.86–0.93)** ^***^

*Note: OR, odds ratio, CI, confidence interval, Data are given as odds ratio (95% confidence interval). Bolded values indicate the significant predictors. Remis = Remission.*

* *p < 0.05,*

** *p < 0.01,*

*** *p < 0.001,*

† *0.05 < p < 0.1*.

## Discussion

In the total sample of the present study, 1,702 students reached the level of clinical insomnia (2.6%). Two months after the outbreak of the pandemic, it was found that the statuses of college students who had clinical insomnia varied: some fully recovered and no longer had insomnia (recovery group, 28%), some showed reduction in symptoms back to mild insomnia (remission group, 26.4%), and nearly a half continued to suffer from probable clinical insomnia (chronic group, 45.7%). Significant between-group differences were found on three aspects of lifestyle: food intake, Internet use, and daily exercise. Further regression analysis showed that lifestyle was a predictor of insomnia change, when the effects of epidemic and other demographic variables were controlled for.

First of all, we found that regular food intake was an important factor that buffers the development of insomnia symptoms, i.e., the remission and recovery groups maintained higher food intake regularity than those with chronic insomnia. The two basic activities of food intake and sleep are rhythmical (18, 64). Regular food intake in terms of time and amount may help better stabilize daily biological rhythms, and develop good sleep hygiene habits to improve the symptoms of insomnia. Existing studies have also found that regular food intake helps avoid circadian clock disorders and reduce metabolic disorders (65). The current study provided evidence for that the regularity of food intake and sleep, which are two basic activities, may have a certain coupling effect. The level of lifestyle regularity was closely associated with the recovery process of insomnia, that is, individuals with more regular lifestyle exhibited greater degree of recovery from insomnia.

In addition, we found that a lack of daily exercise was a risk factor for individuals to develop chronic insomnia or not be able to recover fully, compared with those in the recovery group. According to the WHO, 2.5 h of exercise per week (0.5 h a day multiplied by 5 days) is enough to provide a significant health boost (66). Results of randomized controlled trials (RCT) using exercise up to this dose (2.5 h in total per week) also suggested that regular exercise can help reduce the insomnia symptoms (32–35). This may be because regular exercise can help individuals break the vicious cycle of insomnia (20). The result of the present study further suggested that a longer period of exercise seems to have more benefits, especially more than 60 min of daily exercise, may promote reduction in insomnia, with the hope to recover to normal sleep level. This was similar to the findings of a longitudinal study of college students, which also suggested that 60 min or more of moderate physical activity could help maintain physical and mental health during the pandemic (51). In the case of home isolation during the pandemic, the amount of physical and mental activity, such as daily life, work, and study, is greatly reduced. Thus, students may need even more exercise to maintain the balance between energy intake and expenditure.

The current study found that prolonged Internet use was quite prevalent among Chinese college students from Guangdong province during the COVID-19 epidemic. More than 79.7% of students with insomnia symptoms use the Internet for more than 5 h per day, in another words, prolonged Internet use has been linked to insomnia, which is consistent with a number of studies on Internet overuse, and the overuse or poor use of related electronic products (67–70). The present study also found that more than 5 h of Internet use is a risk factor for chronic insomnia, it has to do with a longitudinal design for mobile excessive use of relation with insomnia results consistent, namely insomnia may be the result of excessive use of network rather than a cause (40). Similarly, two longitudinal studies in Italy found a strong association between smartphone use and insomnia, with increased use of electronic devices within 2 h of bedtime exacerbating insomnia symptoms, and reduced use time improving insomnia symptoms (57, 58). Prolonged use of interactive electronic products may not only cause hyper arousal and interferes with healthy sleep initiation, buy also make deep sleep more difficult (71, 72). Prolonged Internet use and bright lights on screens at night suppress melatonin secretion, forcing circadian rhythms out of whack disrupt the regulation of biological sleep/wake rhythms (67, 69, 73).

It is important to note that the current study did not find a predictive role for Internet use in remission vs. recovery, or persistent insomnia vs. remission. This suggested that Internet use may not be as sensitive as exercise and food intake in the prognosis of insomnia. It is worth noting that this study only investigated the time of Internet use in a day as a whole, without specific time points, which may inevitably limit the sensitivity of the prediction of Internet use duration, considering a strong relationship between the changes of evening exposure to electronic devices and the time course of sleep problems, which have been consistently reported (57, 58). Food intake and exercise, while both being sensitive in predicting the prognosis of insomnia, play different roles. Regular food intake was relevant to a fundamental change in the direction from insomnia to normal sleep. Eating behaviors and sleep behaviors are synchronized that are essentially regulated by interactions between circadian clocks and hormones, and this network of interactions has a strong effect on behavior. This means that these two behaviors are in a multi-layered network system, and changing one factor may affect the whole network. So, maintaining a stable food intake rhythm may provide a way to stabilize circadian rhythms (64, 65). Thus, maintaining stable food intake behavior can help restore the circadian rhythm of the master biological clock, so as to improve insomnia symptoms. In contrast, the effect of daily exercise on the recovery of insomnia are primarily on the magnitude of improvement. Various mechanisms have been proposed to explain how exercise may render sleep benefits. It is postulated that exercise increases energy expenditure and body temperature in a manner that facilitates sleep for recuperation of the body (74–76). In general, it is important to maintain a healthy lifestyle during quarantine in order to help the recovery of insomnia.

## Limitations and Directions for Future Research

The current study was conducted in the context of epidemic home isolation, during which the influence of other external factors on insomnia symptoms was reduced, so it was more conducive to explore the influence of daily lifestyle on insomnia. The setting of quarantines helps control for some variables while also being distinctive to everyday situation. Thus, it should be cautious in extrapolating to the relationship between lifestyle and the development of insomnia in general. More importantly, the development of insomnia and lifestyle were both obtained through self-report observations in the current study, without manipulation of the variable. Therefore, the current study can't rule out the possibility of the opposite relationship, i.e., the improvement in insomnia may leads to a more regular lifestyle, or that it could work both ways. Rigorous RCT experiment is needed to explore the impact of lifestyle on the improvement of insomnia.

Sleep measures was not taken by objective measurements, such as the recognized gold standard PSG (Polysomnography). There were also limitations in the evaluation of lifestyle. Only general surveys were conducted on the three lifestyles, and standardized questionnaires were not employed. Lifestyle details were thus not available, such as the time of the meal, the structure of food intake, and the type of exercise. Future research can conduct a detailed investigation of lifestyle from different aspects, which may be more conducive to the understanding of the relationship between lifestyle and insomnia development, and provide operational guidance for insomnia rehabilitation.

## Conclusion

The regularity of lifestyle is closely related to the recovery of insomnia in home quarantine college students during the COVID-19 epidemic. The food intake regularity in the lifestyle plays the more basic role than exercise, and to some extent, acts as the premise of other aspects of the lifestyle.

## Data Availability Statement

The raw data supporting the conclusions of this article will be made available by the authors, without undue reservation.

## Ethics Statement

The studies involving human participants were reviewed and approved by Human Research Ethics Committees of South China Normal University (Ethics_No._SCNU-PSY-2020-01-001). The patients/participants provided their written informed consent to participate in this study.

## Author Contributions

FF and JZhao: conceptualization. FF and JZhan: methodology. JZhan and LM: formal analysis and writing—original draft preparation. JZhan, ZM, and DW: investigation. JZhan, LM, and HC: data curation. JZhan, JZhao, and FF: writing—review and editing. All authors contributed to the article and approved the submitted version.

## Funding

The present study was funded by National Natural Science Foundation of China (Grant No. 31871129); Research on the Processes and Repair of Psychological Trauma in Youth, Project of Key Institute of Humanities and Social Sciences, MOE (Grant No. 16JJD190001); Guangdong Province Universities and Colleges Pearl River Scholar Funded Scheme (GDUPS 2016); and Graduate Research and Innovation Project of School of Psychology, South China Normal University (PSY-SCNU202017), and Special Funds for the Cultivation of Guangdong College Students' Scientific and Technological Innovation. (Climbing Program Special Funds: pdjh2021a0131).

## Conflict of Interest

The reviewer SL declared a shared affiliation, with one of the authors JZ to the handling editor at the time of the review. The remaining authors declare that the research was conducted in the absence of any commercial or financial relationships that could be construed as a potential conflict of interest.

## Publisher's Note

All claims expressed in this article are solely those of the authors and do not necessarily represent those of their affiliated organizations, or those of the publisher, the editors and the reviewers. Any product that may be evaluated in this article, or claim that may be made by its manufacturer, is not guaranteed or endorsed by the publisher.

## References

[1] Gaultney JF. The prevalence of sleep disorders in college students: impact on academic performance. J Am Coll health. (2010) 59:91–7. 10.1080/07448481.2010.483708 20864434

[2] Ibrahim AK Kelly SJ Adams CE Glazebrook C A. systematic review of studies of depression prevalence in university students. J Psychiatr Res. (2013) 47:391–400. 10.1016/j.jpsychires.2012.11.015 23260171

[3] Tsai LL Li SP. Sleep patterns in college students: gender and grade differences. J Psychosom Res. (2004) 56:231–7. 10.1016/S0022-3999(03)00507-5 15016583

[4] Hicks RA Fernandez C Pellegrini RJ. Striking changes in the sleep satisfaction of university students over the last two decades. Percept Mot Skills. (2001) 93:660. 10.2466/pms.2001.93.3.660 11806582

[5] Sivertsen B Vedaa Ø Harvey AG Glozier N Pallesen S Aarø LE. Sleep patterns and insomnia in young adults: A national survey of Norwegian university students. J Sleep Res. (2019) 28:e12790. 10.1111/jsr.12790 30515935

[6] Jiang XL Zheng XY Yang J Ye CP Chen YY Zhang ZG. systematic review of studies on the prevalence of insomnia in university students. Public Health. (2015) 129:1579–84. 10.1016/j.puhe.2015.07.030 26298588

[7] Ohayon MM Smirne S. Prevalence and consequences of insomnia disorders in the general population of Italy. Sleep Med. (2002) 3:115–120. 10.1016/S1389-9457(01)00158-7 14592229

[8] Xiang YT Ma X Cai ZJ Li SR Xiang YQ Guo HL. The prevalence of insomnia, its sociodemographic and clinical correlates, and treatment in rural and urban regions of Beijing, China: a general population-based survey. Sleep. (2008) 31:1655–62. 10.1093/sleep/31.12.1655 19090321 PMC2603488

[9] Carney CE Moss TG Lachowski AM Atwood ME. Understanding mental and physical fatigue complaints in those with depression and insomnia. Behav Sleep Med. (2014) 12:272–89. 10.1080/15402002.2013.801345 24128300

[10] Fernandez-Mendoza J Vgontzas AN. Insomnia and its impact on physical and mental health. Curr Psychiatry Rep. (2013) 15:418. 10.1007/s11920-013-0418-8 24189774 PMC3972485

[11] Mason EC Harvey AG. Insomnia before and after treatment for anxiety and depression. J Affect Disord. (2014) 168:415–21. 10.1016/j.jad.2014.07.020 25108278

[12] Wong MM Brower KJ. The prospective relationship between sleep problems and suicidal behavior in the National Longitudinal Study of Adolescent Health. J Psychiatr Res. (2012) 46:953–9. 10.1016/j.jpsychires.2012.04.008 22551658 PMC3613125

[13] Liu RT Steele SJ Hamilton JL Do Q Furbish K Burke TA. Sleep and suicide: a systematic review and meta-analysis of longitudinal studies. Clin Psychol Rev. (2020) 81:101895. 10.1016/j.cpr.2020.101895 32801085 PMC7731893

[14] de Zambotti M Goldstone A Colrain IM Baker FC. Insomnia disorder in adolescence: Diagnosis, impact, and treatment. Sleep Med Rev. (2018) 39:12–24. 10.1016/j.smrv.2017.06.009 28974427 PMC5931364

[15] Santiago GTP de Menezes Galvão AC de Almeida RN Mota-Rolim SA Palhano-Fontes F. Maia-de-Oliveira JP. Changes in cortisol but not in brain-derived neurotrophic factor modulate the association between sleep disturbances and major depression. Front Behav Neurosci. (2020) 14:44. 10.3389/fnbeh.2020.00044 32410966 PMC7199815

[16] Mora Ripoll R. Lifestyle medicine: the importance of considering all the causes of disease. Rev Psiquiatr Salud Ment. (2012) 5:48–52. 10.1016/j.rpsm.2011.04.002 22854504

[17] Lianov L. Johnson M. Physician competencies for prescribing lifestyle medicine. JAMA. (2010) 304:202–3. 10.1001/jama.2010.903 20628134

[18] Bechtold DA Loudon AS. Hypothalamic clocks and rhythms in feeding behaviour. Trends Neurosci. (2013) 36:74–82. 10.1016/j.tins.2012.12.007 23333345

[19] Kenny TE Van Wijk M Singleton C Carter JC. An examination of the relationship between binge eating disorder and insomnia symptoms. Eur Eat Disord Rev. (2018) 26:186–96. 10.1002/erv.2587 29542203

[20] Driver HS Taylor SR. Exercise and sleep. Sleep Med Rev. (2000) 4:387–402. 10.1053/smrv.2000.0110 12531177

[21] Baron KG Reid KJ Zee PC. Exercise to improve sleep in insomnia: exploration of the bidirectional effects. J Clin Sleep Med. (2013) 9:819–24. 10.5664/jcsm.2930 23946713 PMC3716674

[22] Chennaoui M Arnal PJ Sauvet F Léger D. Sleep and exercise: a reciprocal issue? Sleep Med Rev. (2015) 20:59–72. 10.1016/j.smrv.2014.06.008 25127157

[23] Cheung LM Wong WS. The effects of insomnia and internet addiction on depression in Hong Kong Chinese adolescents: an exploratory cross-sectional analysis. J Sleep Res. (2011) 20:311–7. 10.1111/j.1365-2869.2010.00883.x 20819144

[24] Guo L Luo M Wang WX Huang GL Xu Y Gao X. Association between problematic Internet use, sleep disturbance, and suicidal behavior in Chinese adolescents. J Behav Addict. (2018) 7:965–75. 10.1556/2006.7.2018.115 30474380 PMC6376369

[25] Binks H. E Vincent G Gupta C Irwin C Khalesi S. Effects of diet on sleep: a narrative review. Nutrients. (2020) 12:936. 10.3390/nu12040936 32230944 PMC7230229

[26] Vardar E Caliyurt O Arikan E Tuglu C. Sleep quality and psychopathological features in obese binge eaters. Stress Health. (2010) 20:35–41. 10.1002/smi.992

[27] Cheng FW Li Y Winkelman JW Hu FB Rimm EB. Gao X. Probable insomnia is associated with future total energy intake and diet quality in men. Am J Clin Nutr. (2016) 104:462–9. 10.3945/ajcn.116.131060 27413124 PMC4962161

[28] Benedict C Brandão LEM Merikanto I Partinen M Bjorvatn B. Cedernaes J. Meal and Sleep Timing before and during the COVID-19 Pandemic: A Cross-Sectional Anonymous Survey Study from Sweden. Clocks Sleep. (2021) 3:251–8. 10.3390/clockssleep3020015 33921946 PMC8167780

[29] Zhu S Zhang X Ma W Zhang S Wu S Gao X. The association between insomnia and night eating behavior in Chinese adults. Curr Develop Nutr. (2020) 4:168. 10.1093/cdn/nzaa046_08032294828

[30] Kandeger A Egilmez U Sayin AA Selvi Y. The relationship between night eating symptoms and disordered eating attitudes *via* insomnia and chronotype differences. Psychiatry Res. (2018) 268:354–7. 10.1016/j.psychres.2018.08.003 30098543

[31] Buman MP Phillips BA Youngstedt SD Kline CE Hirshkowitz M. Does nighttime exercise really disturb sleep? Results from the 2013 National Sleep Foundation Sleep in America Poll. Sleep Med. (2014) 15:755–61. 10.1016/j.sleep.2014.01.008 24933083

[32] King AC Pruitt LA Woo S Castr CM Ahn DK Vitiello MV. Effects of moderate-intensity exercise on polysomnographic and subjective sleep quality in older adults with mild to moderate sleep complaints. J Gerontol A Biol Sci Med Sci. (2008) 63:997–1004. 10.1093/gerona/63.9.997 18840807 PMC7182081

[33] Hartescu I Morgan K Stevinson CD. Increased physical activity improves sleep and mood outcomes in inactive people with insomnia: a randomized controlled trial. J Sleep Res. (2015) 24:526–34. 10.1111/jsr.12297 25903450

[34] Tan X Alén M. Wiklun P Partinen M Cheng S. Effects of aerobic exercise on home-based sleep among overweight and obese men with chronic insomnia symptoms: a randomized controlled trial. Sleep Med. (2016) 25:113–21. 10.1016/j.sleep.2016.02.010 27823703

[35] Chen LJ Fox KR Ku PW Chan YW Effects Effects of aquatic exercise on sleep in older adults with mild sleep impairment: a randomized controlled trial. Int J Behav Med. (2016) 23:501–6. 10.1007/s12529-015-9492-0 26025630

[36] Lowe H Haddock G Mulligan LD Gregg L Fuzellier-Hart A Carter LA. Does exercise improve sleep for adults with insomnia? A systematic review with quality appraisal. Clin Psychol Rev. (2019) 68:1–12. 10.1016/j.cpr.2018.11.002 30617012

[37] Thomala LL. Number of Internet Users in China 2017-2023. (2020). Available at: https://www.statista.com/statistics/278417/number-of-internet-users-in-china/

[38] Lam LT. Internet gaming addiction, problematic use of the internet, and sleep problems: a systematic review. Curr Psychiatry Rep. (2014) 16:444. 10.1007/s11920-014-0444-1 24619594

[39] Shen Y Wang L Huang C Guo J De Leon SA Lu J. Sex differences in prevalence, risk factors and clinical correlates of internet addiction among Chinese college students. J Affect Disord. (2021) 279:680–6. 10.1016/j.jad.2020.10.054 33190119

[40] Liu S Wing YK Hao Y Li W Zhang J Zhang B. The associations of long-time mobile phone use with sleep disturbances and mental distress in technical college students: a prospective cohort study. Sleep. (2019) 42. 10.1093/sleep/zsy213 30395300

[41] Chen R Chou KR Huang YJ Wang TS Liu SY Ho LY. Effects of a SARS prevention programme in Taiwan on nursing staff's anxiety, depression and sleep quality: a longitudinal survey. Int J Nurs Stud. (2006) 43:215–25. 10.1016/j.ijnurstu.2005.03.006 15927185 PMC7094227

[42] Johal SS. Psychosocial impacts of quarantine during disease outbreaks and interventions that may help to relieve strain. N Z Med J. (2009) 122:47–52. 19652680

[43] Rossi R Socci V Talevi D Mensi S Niolu C Pacitti F. COVID-19 Pandemic and Lockdown Measures Impact on Mental Health Among the General Population in Italy. Front Psychiatry. (2020) 11:790. 10.3389/fpsyt.2020.00790 32848952 PMC7426501

[44] Huang Y Zhao N. Generalized anxiety disorder, depressive symptoms and sleep quality during COVID-19 outbreak in China: a web-based cross-sectional survey. Psychiatry Res. (2020) 288:112954. 10.1016/j.psychres.2020.112954 32325383 PMC7152913

[45] Kokou-Kpolou CK Megalakaki O Laimou D Kousouri M. Insomnia during COVID-19 pandemic and lockdown: Prevalence, severity, and associated risk factors in French population. Psychiatry Res. (2020) 290:113128. 10.1016/j.psychres.2020.113128 32563951 PMC7255302

[46] Voitsidis P Gliatas I Bairachtari V Papadopoulou K Papageorgiou G Parlapani E. Insomnia during the COVID-19 pandemic in a Greek population. Psychiatry Res. (2020) 289:113076. 10.1016/j.psychres.2020.113076 32434093 PMC7217074

[47] Morin CM Bjorvatn B Chung F Holzinger B Partinen M Penzel T. Insomnia, anxiety, and depression during the COVID-19 pandemic: an international collaborative study. Sleep Med. (2021) 87:38–45. 10.1016/j.sleep.2021.07.035 34508986 PMC8425785

[48] Kay-Stacey M Attarian H. Advances in the management of chronic insomnia. BMJ. (2016) 354:i2123. 10.1136/bmj.i2123 27383400

[49] Viselli L Salfi F D'Atri A Amicucci G Ferrara M. Sleep Quality, Insomnia Symptoms, and Depressive Symptomatology among Italian University Students before and during the Covid-19 Lockdown. Int J Environ Res Public Health. (2021) 18:13346. 10.3390/ijerph182413346 34948954 PMC8705602

[50] Di Renzo L Gualtieri P Cinelli G Bigioni G Soldati L Attinà A. Psychological aspects and eating habits during COVID-19 home confinement: results of EHLC-COVID-19 Italian Online Survey. Nutrients. (2020) 12:2152. 10.3390/nu12072152 32707724 PMC7401000

[51] Balanzá-Martínez V Kapczinski F de Azevedo Cardoso T Atienza-Carbonell B Rosa AR Mota JC. The assessment of lifestyle changes during the COVID-19 pandemic using a multidimensional scale. Rev Psiquiatr Salud Ment (Engl Ed). (2021) 14:16–26. 10.1016/j.rpsm.2020.07.003 32962948 PMC10068027

[52] Zhang Y Zhang H Ma X Di Q. Mental Health Problems during the COVID-19 Pandemics and the Mitigation Effects of Exercise: A Longitudinal Study of College Students in China. Int J Environ Res Public Health. (2020) 17:3722. 10.3390/ijerph17103722 32466163 PMC7277113

[53] Lu C Chi X Liang K Chen ST Huang L Guo T. Moving More and Sitting Less as Healthy Lifestyle Behaviors are Protective Factors for Insomnia, Depression, and Anxiety Among Adolescents During the COVID-19 Pandemic. Psychol Res Behav Manag. (2020) 13:1223–33. 10.2147/PRBM.S284103 33364864 PMC7751784

[54] Davy JP Scheuermaier K Roden LC. Christi CJ Bentle A Gomez-Olive FX. The COVID-19 Lockdown and Changes in Routine-Oriented Lifestyle Behaviors and Symptoms of Depression, Anxiety, and Insomnia in South Africa. J Phys Act Health. (2021) 18:1046–57. 10.1123/jpah.2020-0863 34186512

[55] DATAREPORTAL. Digital 2020: April Global Statshot. Available at: https://datareportal.com/reports/digital-2020-april-global-statshot (accessed September 1, 2020).

[56] Ofcom. Lockdown Leads to Surge in TV Screen Time and Streaming. Available at: https://www.ofcom.org.uk/about-ofcom/latest/features-and-news/lockdown-leads-to-surge-in-tv-screen-time-and-streaming (accessed September 1st, 2020).

[57] Salfi F D'Atri A Tempesta D Ferrara M. Sleeping under the waves: A longitudinal study across the contagion peaks of the COVID-19 pandemic in Italy. J Sleep Res. (2021) 30:e13313. 10.1111/jsr.13313 33687798 PMC8250209

[58] Salfi F Amicucci G Corigliano D D'Atri A Viselli L Tempesta D. Changes of evening exposure to electronic devices during the COVID-19 lockdown affect the time course of sleep disturbances. Sleep. (2021) 44:zsab080. 10.1093/sleep/zsab080 34037792 PMC8194574

[59] Chen P Mao L Nassis GP Harmer P Ainsworth BE Li F. Coronavirus disease (COVID-19): The need to maintain regular physical activity while taking precautions. J Sport Health Sci. (2020) 9:103–4. 10.1016/j.jshs.2020.02.001 32099716 PMC7031771

[60] Balanzá-Martínez V Atienza-Carbonell B Kapczinski F De Boni RB. Lifestyle behaviours during the COVID-19 time to connect. Acta Psychiatr Scand. (2020) 141:399–400. 10.1111/acps.13177 32324252 PMC7264786

[61] Ma Z Zhao J Li Y Chen D Wang T Zhang Z. Mental health problems and correlates among 746 217 college students during the coronavirus disease 2019 outbreak in China. Epidemiol Psychiatr Sci. (2020) 29:e181. 10.1017/S2045796020000931 33185174 PMC7681173

[62] Li Y Zhao J Ma Z McReynolds LS Lin D Chen. Mental Health Among College Students During the COVID-19 Pandemic in China: A 2-Wave Longitudinal Survey. J Affect Disord. (2021) 281:597–604. 10.1016/j.jad.2020.11.109 33257043

[63] Liu X Yang Y Liu ZZ Luo Y Fan F Jia CX. Psychometric properties of youth self-rating insomnia scale (YSIS) in Chinese adolescents. Sleep Biol Rhythms. (2019) 17:339–48. 10.1007/s41105-019-00222-3

[64] Koop S Oster H. Eat, sleep, repeat-endocrine regulation of behavioral circadian rhythms. FEBS J. (2021). 10.1111/febs.16109 34228879

[65] Challet E. The circadian regulation of food intake. Nat Rev Endocrinol. (2019) 15:393–405. 10.1038/s41574-019-0210-x 31073218

[66] Global Recommendations on Physical Activity for Health. Geneva: World Health Organization (2010). Available at: http://apps.who.int/iris/bitstream/handle/10665/44399/9789241599979_eng.pdf;sequence=1 26180873

[67] Voelker R. Stress sleep loss, and substance abuse create potent recipe for college depression. JAMA. (2004) 291:2177–9. 10.1001/jama.291.18.2177 15138228

[68] Choi K Son H Park M Han J Kim K Lee B. Internet overuse and excessive daytime sleepiness in adolescents. Psychiatry Clin Neurosci. (2009) 63:455–62. 01925.x 10.1111/j.1440-1819.2009.01925.x 19490510

[69] Gradisar M Wolfson AR Harvey AG Hale L Rosenberg R Czeisler CA. The sleep and technology use of Americans: findings from the National Sleep Foundation's 2011 Sleep in America poll. J Clin Sleep Med. (2013) 9:1291–9. 10.5664/jcsm.3272 24340291 PMC3836340

[70] Do KY Lee KS. Relationship between problematic internet use, sleep problems, and oral health in Korean Adolescents: A National Survey. Int J Environ Res Public Health. (2018) 15:1870. 10.3390/ijerph15091870 30158492 PMC6164655

[71] Kaneita Y Ohida T Osaki Y Tanihata T Minowa M. Suzuki K. Kaneita Y Ohida T Osaki Y. Insomnia among Japanese adolescents: a nationwide representative survey. Sleep. (2006) 29:1543–50. 10.1093/sleep/29.12.1543 17252885

[72] Suganuma N Kikuchi T Yanagi K Yamamura S Morishima H Adachi H. Using electronic media before sleep can curtail sleep time and result in self-perceived insufficient sleep. Sleep Biol Rhythms. (2010) 5:204–14. 10.1111/j.1479-8425.2007.00276.x

[73] Honda M Genba M Kawakami J Nishizono-Maher A. A sleep and life-style survey of Japanese high school boys: factors associated with frequent exposure to bright nocturnal light. Sleep Biol Rhythms. (2008) 6:110–19. 10.1111/j.1479-8425.2008.00340.x

[74] McGinty D. Szymusiak R. Keeping cool: a hypothesis about the mechanisms and functions of slow-wave sleep. Trends Neurosci. (1990) 13:480–7. 10.1016/0166-2236(90)90081-K 1703678

[75] Adam K. Oswald I. Protein synthesis, bodily renewal and the sleep-wake cycle Clin Sci (Lond). (1983) 65:561–7. 10.1042/cs0650561 6194928

[76] Berger RJ. Phillips NH. Comparative aspects of energy metabolism, body temperature and sleep Acta Physiol Scand Suppl. (1988) 574:21–7. 3072836

